# Galahad: a web server for drug effect analysis from gene expression

**DOI:** 10.1093/nar/gkv436

**Published:** 2015-05-04

**Authors:** Griet Laenen, Amin Ardeshirdavani, Yves Moreau, Lieven Thorrez

**Affiliations:** 1 Department of Electrical Engineering (ESAT), STADIUS Center for Dynamical Systems, Signal Processing and Data Analytics, KU Leuven, Leuven, 3001, Belgium; 2 iMinds Medical IT Department, KU Leuven, Leuven, 3001, Belgium; 3 Interdisciplinary Research Facility Life Sciences, Department of Development and Regeneration, KU Leuven Kulak, Kortrijk, 8500, Belgium

## Abstract

*Galahad* (https://galahad.esat.kuleuven.be) is a web-based application for analysis of drug effects. It provides an intuitive interface to be used by anybody interested in leveraging microarray data to gain insights into the pharmacological effects of a drug, mainly identification of candidate targets, elucidation of mode of action and understanding of off-target effects. The core of *Galahad* is a network-based analysis method of gene expression. As an input, *Galahad* takes raw *Affymetrix* human microarray data from treatment versus control experiments and provides quality control and data exploration tools, as well as computation of differential expression. Alternatively, differential expression values can be uploaded directly. Using these differential expression values, drug target prioritization and both pathway and disease enrichment can be calculated and visualized. Drug target prioritization is based on the integration of the gene expression data with a functional protein association network. The web site is free and open to all and there is no login requirement.

## INTRODUCTION

The pharmaceutical industry is facing unprecedented productivity challenges. Only one out of 20 compounds that entered clinical trials in 2006–2008 made it to become a marketed product (1,2). Causes of failure change during the course of development. Early in the process, compounds fail primarily for safety reasons. Compounds that successfully navigate Phase 1 increasingly drop out due to lack of efficacy in Phases 2 and 3 (3). With safety and efficacy being the main bottlenecks, a better knowledge of a candidate drug's mode of action and its off-target effects could be of substantial value to drug development.

DNA microarray technology enables genome-wide analysis of the transcriptional response to a compound treatment, which is frequently employed to study the effects of small molecules on cells (4). Gene expression patterns that describe the perturbation of a biological system by a drug compound can provide valuable information for identifying compound–protein interactions and resulting effects (5) prior to clinical trials. In addition, this information may also be useful for already marketed drugs, in light of drug repositioning (6). This approach has proven to be successful as demonstrated by the many applications of the *Connectivity Map* (7–10).

We have developed an easy-to-use web server called *Galahad*, for the in-depth exploration of a drug's mode of effect based on gene expression changes following treatment. Our software provides multiple tools needed for gaining new insights into the biological effects of a drug by combining *Affymetrix* human gene expression data preprocessing, quality assessment and exploratory analysis, genome-wide drug target prioritization, differential expression analysis and pathway, as well as disease phenotype enrichment. Drug target prioritization relies on the integration of the obtained differential expression values with prior knowledge on functional protein associations. By means of a network neighborhood analysis the functional relation between proteins is taken into account, which significantly improves the expression-based prediction of drug–protein interactions.

## INPUT

The submission page is easy to find and help is available by mouse-over where input is required from the user. The main input for *Galahad* are raw DNA microarray data derived from both untreated control samples and samples treated with a drug of interest, with one of the three common *Affymetrix GeneChips* HG-U133A, HG-U133 Plus 2.0 or HuGene 1.0 ST. Both the control and the treatment data need to be uploaded as a zipped file containing .CEL files for at least two samples. The total size of both these zip files is currently limited to 250 MB. Once the data are uploaded completely, *Galahad* can be launched by clicking the submit button. A typical workflow then starts with the preprocessing of these uploaded expression data, either with the RMA (11) or the MAS5.0 algorithm, as indicated by the user. Next, the desired analyses can be selected; these may include quality assessment and exploratory analysis of the data, differential expression analysis, drug target prioritization, and both pathway and disease enrichment. Drug target prioritization and enrichment analysis can also be performed on differential expression data derived from other platforms through the submission of a plain text file containing *HGNC* gene symbols, log_2_ ratios and corresponding *P*-values. The input interface provides the possibility to enter a study name, as well as an email address to get notified when all computations are finished.

## METHODS

**Figure 1.**
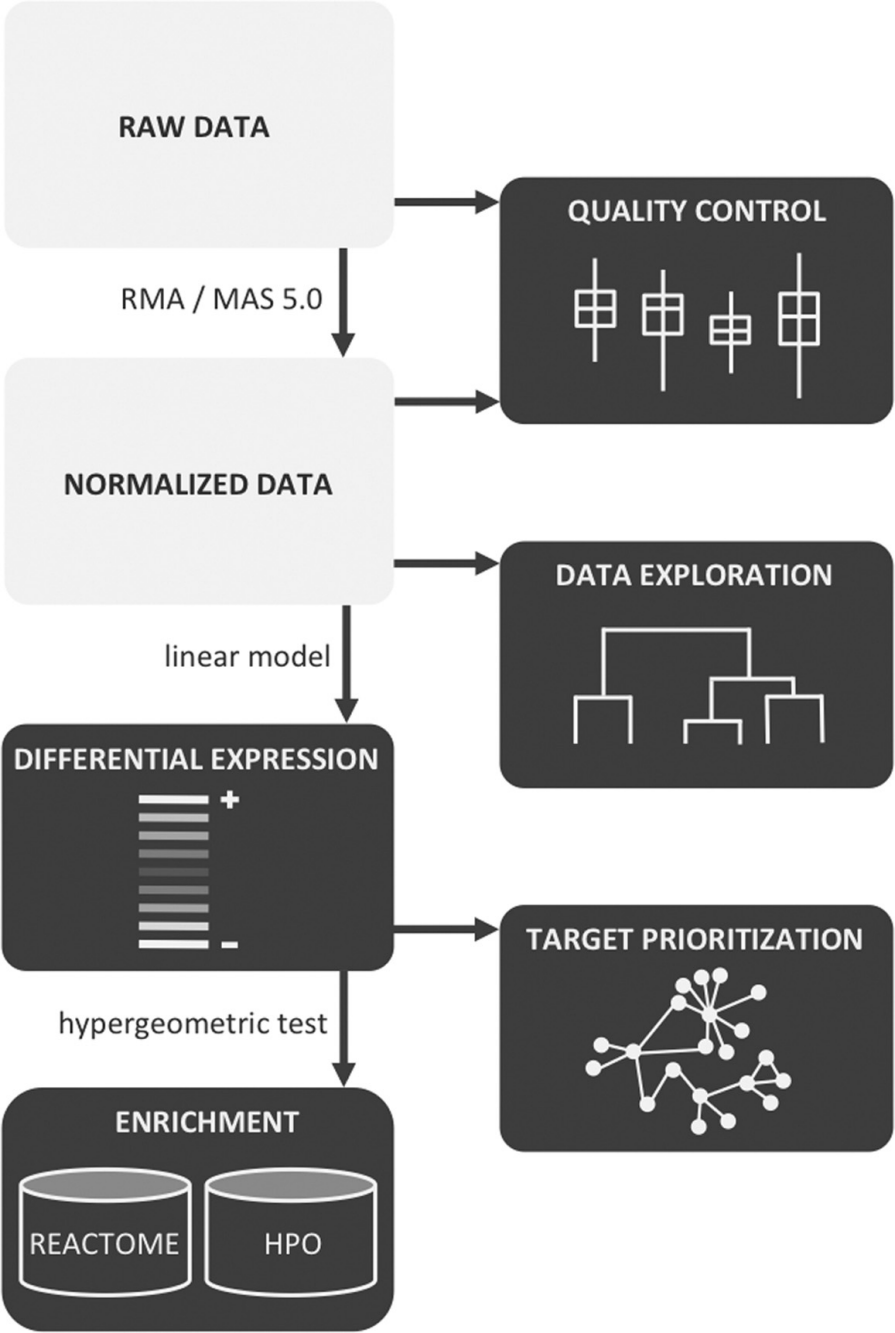
Overview of the different *Galahad* analysis steps. Quality control is performed on both the raw and MAS 5.0 normalized data. After normalization some further exploratory analysis is performed and differential expression is computed. The obtained differential expression values are then used for both pathway and disease phenotype enrichment as well as for network-based drug target prioritization.

An overview of the different analysis steps is depicted in Figure 1.

### Quality control

To assess the quality of the different arrays within the microarray data set, uncover experimental problems and help in deciding whether certain arrays need to be considered as outlying, several quality metrics can be generated. This quality control is mainly based on the *Bioconductor* ArrayTools package, but also relies on functionalities from the affy (12), oligo (13), oligoClasses, genefilter, simpleaffy (14), affyPLM (15) and RColorBrewer libraries. The following plots are produced: side-by-side boxplots of the log intensities to assess the overall signal comparability, probe level model-based pseudo-images to detect spatial biases and a NUSE and RLE plot to check for probe set homogeneity. In addition, for HG-U133A and HG-U133 Plus 2.0 arrays, the average background, the percentage of genes called present and the intensities of the spike-in hybridization controls give insight into the hybridization quality. Scale factors provide information on signal comparability and 3′/5′ ratios for β-actin and GAPDH can be used to assess sample quality. Finally, an RNA degradation plot is generated for these array types.

### Data exploration

To assess the correlation and grouping of samples three more plots are generated on the preprocessed data: a PCA plot, a hierarchical clustering and an intensity correlation heat map.

### Differential expression

Differential expression analysis to determine the significance of gene up- and downregulation following drug treatment is based on the *Bioconductor* limma (16) package. A linear model is fitted to the expression data for each gene and by computing a global variance estimator using empirical Bayes moderated *t*-statistics and corresponding *P*-values are obtained. These *P*-values are then corrected for multiple testing by the method of Benjamini and Hochberg (17), by controlling the false discovery rate (FDR).

### Drug target prioritization

**Figure 2.**
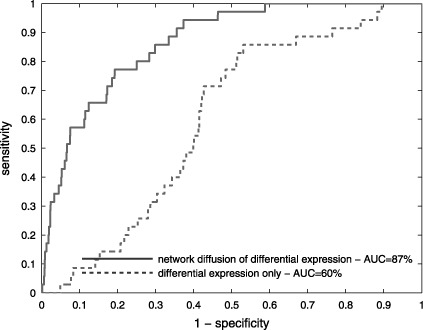
ROC curves comparing the performance of our network diffusion-based drug target prioritization algorithm and prioritization based on differential expression only on a new validation data set consisting of 35 drug treatments corresponding to 85 known targets.

Genome-wide drug target prioritization is performed by means of an in-house developed algorithm for network neighborhood analysis (18) integrating the expression data with functional protein association information derived from *STRING* (19). More specifically, this approach prioritizes potential targets by diffusing the differential expression signals to neighboring network nodes based on the confidence scores of these functional associations. *P-*values are obtained by random reassignment of the expression values to the network nodes and computation of the corresponding randomized scores for all genes. The method has now been validated on a new test set consisting of 35 publicly available *Affymetrix* gene expression data sets where treatment with a chemical compound was tested against a control. In *ChEMBL* (20), we identified all proteins interacting with an IC_50_ below 10 μM with one of these compounds, resulting in a total of 85 distinct targets. A table with the 35 GEO accession numbers, together with the drugs of treatment and their targets as derived from *ChEMBL*, is provided as supplementary file S1. Based on the positions of these known targets in the protein rankings obtained with our method for each of the interacting compounds, an AUC of 87% was achieved (Figure 2). In comparison, ranking the genes based on differential expression data only resulted in an AUC of 60%.

### Enrichment

Functional enrichment of differentially expressed genes is performed by means of a hypergeometric test, followed by an FDR-based multiple testing correction of the obtained *P-*values. In this way, *Reactome* (21) pathways involved in the drug's mode of effect are predicted, as well as associated disease phenotypes from the *Human Phenotype Ontology* (HPO) (22) enabling side effect prediction and drug repositioning.

## OUTPUT

After submission the user is automatically redirected to the results page, where the job details are provided. When the job is completed, the results appear on this page, and an email containing a hyperlink to the page is sent to the user if a valid email address was provided. If cookies are enabled, a hyperlink to the result page (study name may help in case of submitting several jobs) is also stored in a personal history for 20 days. The output is displayed in a series of tabs corresponding to the different analyses selected by the user.

### Quality control and data exploration

In the Quality Control and Data Explorations tabs, the different plots are displayed along with a table in which each sample is assigned a new index as used in the plots. By clicking on a plot a larger image is shown, together with a short explanation.

### Differential expression

The Differential Expression tab contains a table listing all genes together with their log_2_ ratios and both the naive and adjusted *P*-values for differential expression. Genes are ranked according to the adjusted *P*-value, but can for example also be ranked according to log_2_ ratio by simply clicking on the respective column header. Furthermore, different filters can be applied. The complete table can be exported in comma-separated value (CSV) format, which can be imported by any spreadsheet. By clicking on a gene name, the user is brought to the corresponding *GeneCards* section. To select the differentially expressed genes used for Enrichment Analysis, a *P*-value threshold can be entered at the top of the page. By default an adjusted *P*-value of 0.01 is used.

### Drug target prioritization

In the Drug Target Prioritization tab, a ranked list of genes as potential targets of the drug can be found together with the network diffusion-based scores and both the naive and adjusted *P*-values for prioritization. Genes of interest can be detected in this list with the aid of the filter function. The complete table can again be exported as a CSV file and clicking on a gene name opens the corresponding *GeneCards* section. In addition, a network-based visualization is available for each gene, showing the 10 interaction partners contributing most to the gene's ranking. Genes in this network are colored according to fold change and sized according to the significance (*t*-statistic) of this differential expression. Clicking on a node or edge opens the corresponding gene or interaction in *STRING*.

### Enrichment

After selecting the differential expression threshold on which the gene set used for enrichment analysis is based, two separate tabs display tables summarizing the enrichment results for Pathway and Disease Enrichment. These tables contain *Reactome* or HPO IDs and names, together with the number of differentially expressed genes in the corresponding gene sets and the accompanying *P*-values and adjusted *P*-values. The differentially expressed genes for each pathway or disease phenotype are accessible through dropdown tables. Clicking on an ID brings the user to the *Reactome* diagram or HPO page. The enrichment tables can again be sorted, filtered and exported as CSV files. In addition, network graphs are available, consisting of the top 10 most significant pathways or disease phenotypes, along with their associated genes colored according to fold change.

## IMPLEMENTATION

The *Galahad* server has three modules: a user interface, a job controller and an analyzer. The user interface provides a database-related service based on a three-tier architecture using *Microsoft .Net* technology. At the presentation layer on top, we developed a user-friendly and rich interface. To speed up interaction between the clients and the server, we added *AJAX* functionalities to retrieve large amounts of data as fast as possible. All the procedures for importing data into and retrieving data from the database are located in the data layer. The business layer represents the main link between the presentation layer and the data layer and thus contains all the rules for processing information.

The job controller is a simple *Java* application responsible for communication between the web application and the analyzer. After submission of a new request the job controller transfers the uploaded control and treatment file to a *Linux* server and runs the analyzer to process the request. When the results are ready the job controller transfers them back to the web server and makes them available for the user.

The analyzer is the main module responsible for analysis of the user's request. This module is completely developed in R. The time needed for a job to finish is depending on the data size, the requested analyses and the workload of our server. Data produced by the analyzer module are stored on a *Microsoft SQL* server. *Galahad* can be accessed with all main web browsers. Uploaded data and the results of analysis are kept private and not viewable by other users through the use of a secure server and are deleted after two weeks. The web site is free and open to all and there is no login requirement.

## EXAMPLE

An example data set is provided on the web site. Results can be directly accessed or input data are provided and can be uploaded. A manual and an example video are also available on the home page.

## CONCLUSION AND OUTLOOK

*Galahad* is an open and free platform to analyze microarray data for the characterization of drug effects. It provides an easy interface where in an integrated way quality control, data exploration, differential expression and both pathway and disease enrichment can be calculated and visualized. In addition, drug target prioritization provides a ranked list of target candidates.

Toward the future, the platform can be extended to take data from RNA sequencing experiments and more complicated experimental designs such as multiple groups and time series, as well as data from other organisms such as mouse and rat. We are also in the process of implementing the network analysis tools from *Galahad* as a standalone R package.

## SUPPLEMENTARY DATA

Supplementary Data are available at NAR Online.

SUPPLEMENTARY DATA
