# Supplementary material for: Galahad: a web server for drug effect analysis from gene expression
Source: Nucleic Acids Res. 2015 May 4;43(Web Server issue):W208–12. doi: 10.1093/nar/gkv436 (PMC4489261; doi:10.1093/nar/gkv436)
Supplement: SUPPLEMENTARY DATA [file supp_43_W1_W208__index.html]

Galahad: a web server for drug effect analysis from gene expression — Galahad: a web server for drug effect analysis from gene expression — SUPPLEMENTARY DATA 

# Galahad: a web server for drug effect analysis from gene expression

## SUPPLEMENTARY DATA

**Files in this Data Supplement:**

- SUPPLEMENTARY DATA
